# Deep normative modelling reveals insights into early-stage Alzheimer's disease using multi-modal neuroimaging data

**DOI:** 10.1186/s13195-025-01753-3

**Published:** 2025-05-15

**Authors:** Ana Lawry Aguila, Luigi Lorenzini, Mohammed Janahi, Frederik Barkhof, Andre Altmann

**Affiliations:** 1 Department of Medical Physics and Biomedical Engineering, UCL Hawkes Institute, University College London (UCL), London, UK; 2 Department of Radiology and Nuclear Medicine, Amsterdam University Medical Center, Amsterdam, 1081 HV The Netherlands; 3 Brain Imaging, Amsterdam, the Netherlands; 4 Medical and Population Genomics Lab, Human Genetics Department, Research Branch, Sidra Medicine, Doha, Qatar; 5 UCL Queen Square Institute of Neurology, University College London, London, WC1 N 3BG UK; 6 Present Address: Athinoula A. Martinos Center for Biomedical Imaging, Harvard Medical School and Massachusetts General Hospital, Boston, USA

**Keywords:** Alzheimer's disease, Normative modelling, MRI, Deep-learning

## Abstract

**Background:**

Exploring the early stages of Alzheimer's disease (AD) is crucial for timely intervention to help manage symptoms and set expectations for affected individuals and their families. However, the study of the early stages of AD involves analysing heterogeneous disease cohorts which may present challenges for some modelling techniques. This heterogeneity stems from the diverse nature of AD itself, as well as the inclusion of undiagnosed or ‘at-risk’ AD individuals or the presence of comorbidities which differentially affect AD biomarkers within the cohort. Normative modelling is an emerging technique for studying heterogeneous disorders that can quantify how brain imaging-based measures of individuals deviate from a healthy population. The normative model provides a statistical description of the ‘normal’ range that can be used at subject level to detect deviations, which may relate to pathological effects.

**Methods:**

In this work, we applied a deep learning-based normative model, pre-trained on MRI scans in the UK Biobank, to investigate ageing and identify abnormal age-related decline. We calculated deviations, relative to the healthy population, in multi-modal MRI data of non-demented individuals in the external EPAD (ep-ad.org) cohort and explored these deviations with the aim of determining whether normative modelling could detect AD-relevant subtle differences between individuals.

**Results:**

We found that aggregate measures of deviation based on the entire brain correlated with measures of cognitive ability and biological phenotypes, indicating the effectiveness of a general deviation metric in identifying AD-related differences among individuals. We then explored deviations in individual imaging features, stratified by cognitive performance and genetic risk, across different brain regions and found that the brain regions showing deviations corresponded to those affected by AD such as the hippocampus. Finally, we found that ‘at-risk’ individuals in the EPAD cohort exhibited increasing deviation over time, with an approximately 6.4 times greater t-statistic in a pairwise t-test compared to a ‘super-healthy’ cohort.

**Conclusion:**

This study highlights the capability of deep normative modelling approaches to detect subtle differences in brain morphology among individuals at risk of developing AD in a non-demented population. Our findings allude to the potential utility of normative deviation metrics in monitoring disease progression.

**Supplementary Information:**

The online version contains supplementary material available at 10.1186/s13195-025-01753-3.

## Introduction

Alzheimer's disease (AD) is the most common form of dementia, accounting for approximately 60–70% of all dementia cases [1]. Pathological changes related to AD can occur decades before symptom onset, but often go undiagnosed for years [2]. Early diagnosis of AD is crucial due to the progressive and irreversible nature of the disease. Detecting AD at its initial stages allows for better management of symptoms, setting expectations for affected individuals and their families, and will help in designing better clinical trials for potential treatments.

Research has led to a multitude of biomarkers and clinical factors used to detect, monitor, and understand the underlying pathology in AD. These include genetic and environmental risk factors [3], cognitive tests, age of onset, symptom profile [4], cerebrospinal fluid (CSF) readouts [5], blood-based markers [6], and neuroimaging-based measures quantifying buildup of proteins [7] and neurodegeneration or atrophy profiles [8]. However, many research studies have found substantial differences in these factors between patients [4, 5, 9]. This heterogeneity may manifest as different subtypes with distinct cognitive trajectories and disease progression [10]. The case–control approach taken in many AD studies may oversimplify this heterogeneity by grouping individuals into broad categories, failing to capture the clinical and biological differences in AD. As such, a shift is needed towards modelling approaches that reflect patient heterogeneity in AD.

Neuroimaging is an invaluable tool for understanding the brain. Structural imaging, in particular, plays a fundamental role in unravelling the complexities of neurodegeneration. As such, several studies using structural T1-weighted MRI have identified both macrostructural changes (e.g., general atrophy) and specific brain regions which exhibit neurodegeneration (e.g., the amygdala and hippocampus) [11]. Diffusion tensor imaging (DTI) metrics, which can measure microstructural white-matter properties in the brain, offer additional, and possibly earlier, biomarkers of neurodegeneration [12]. For example, Tranfa et al. [13] found that AD pathology was associated with white-matter integrity in several tracts. Nir et al. [14] also found that DTI Fractional Anisotropy (FA) and diffusivity measures were correlated with clinical cognitive scores and identified several white-matter regions implicated in AD. However, bar a few commonly seen alterations, these neuroimaging markers show heterogeneous profiles in AD patients, exhibiting a high degree of variability between different patients and disease stages. For example, several atrophy subtypes been identified with distinct symptom profiles and expected trajectories of cognitive decline [15, 16]. Additionally, copathologies have been shown to differentially affect these white-matter markers [13]. With this in mind, we aim to design a study to account for this heterogeneity in neuroimaging features and explore differences in AD biomarkers at an individual level.

Normative modelling is a popular method for describing the ‘normal’ behaviour or expected trajectory of a healthy population which can be used at subject level to detect deviations relating to a disease. These models consider covariates such as age and sex such that we can assess the value of a biomedical feature in relation to the expected value for a healthy individual with a specific set of covariate values. Normative models assume disease cohorts sit at the tails of a healthy population distribution and quantify the distance of an individual from healthy brain patterns, i.e., a deviation value. As such, in the case of AD, we expect individuals with higher cognitive and functional decline to have higher deviation values, i.e., the value of a biomedical feature is substantially higher or lower than expected for a given age and sex. Traditional normative approaches involve learning one normative model per imaging derived feature by training a regression model, for example Gaussian Process Regression (GPR), to predict each biomedical feature from a set of clinical covariates. Such models have been widely used in various disciplines such as psychiatry, psychology, neurology and neuroscience [17] to address and explore disease heterogeneity. The parameters of a normative model are learnt such that they characterise healthy brains from a control population and provide a statistical measure of normality. Thus, applying the normative model to a disease cohort allows for quantification of the deviation, given the demographic covariates, of subjects within this cohort from the norm [18]. However, these traditional approaches consider and derive a deviation measure for each biomarker independently and do not consider the interactions between features.

Recently, deep learning approaches to normative modelling have been proposed to model the interaction across multiple brain imaging features or imaging modalities and derive an aggregate measure of deviation from a multivariate normative distribution [19–21]. These approaches use autoencoders and either incorporate demographic covariates into the modelling framework or adjust for them beforehand. Autoencoders are deep learning models consisting of an encoder network, which maps the input data to a lower-dimensional latent space, and a decoder network, which maps from the latent space back up to the feature space. Here, we use a multi-modal autoencoder [22, 23] as a deep normative model for measuring deviations across multiple features and modalities.

In this work, we explore the potential of such deep normative modelling approaches to offer useful insights into cohorts at risk of developing AD. We applied this approach to T1 and diffusion MRI (dMRI) features from the European Prevention of Alzheimer’s Dementia (EPAD) cohort, comprising healthy individuals recruited as a ‘probability spectrum'population, covering the entire range of anticipated probabilities for Alzheimer’s dementia development [24]. Although some participants are likely to develop AD in the future, at baseline, none of the individuals have an AD diagnosis. As such, the EPAD dataset presents an ideal opportunity for investigating the early stages of AD by exploring AD related biomarkers before any signs of cognitive decline become evident. In addition to the heterogeneity inherent to AD, as the EPAD dataset covers the full spectrum of disease risk, it is likely to be particularly diverse due to a mix of disease labels. As such normative modelling, which is disease agnostic, is particularly apt for modelling the EPAD dataset. Specifically, we aimed to:

• Evaluate the degree to which general and AD-specific normative deviation metrics correlate with AD-related biomarkers and cognitive performance within a non-demented at-risk cohort.
• Assess the extent of neuroanatomical variability between individuals based on patterns of outliers across different levels of cognitive performance and genetic risk.
• Assess whether deviation measures align with expectations over time in healthy and ‘at-risk’ cohorts.

## Methods

### Data processing

Here, we worked with two datasets; the UK Biobank to train our normative model on healthy brain variation, and the EPAD dataset as the external target dataset. To pre-train the model, we used 12,844 healthy subjects from the UK Biobank. Our cohort was smaller than the total number of subjects with imaging data due to the date of access (accessed 26/10/2019), restricting to healthy sex-matched controls, and selecting individuals for which both T1 and dMRI features were available. This research has been conducted using the UK Biobank Resource under Application Number 70047. Subjects were selected such that they had no neurological, psychiatric disorders or head trauma according to available ICD9 and ICD10 codes. We used pre-processed (provided by the UK Biobank [25]) FreeSurfer grey-matter volumes for 66 cortical (Desikan-Killiany atlas) and 16 subcortical brain regions, and Fractional Anisotropy (FA) and Mean Diffusivity (MD) measurements for 32 white matter tracts (John Hopkins University atlas) as the input features to the normative model. Demographic information for the UK Biobank cohort is provided in Supplementary Table S1.

For model analysis we used the EPAD dataset with a total of 929 subjects. We split this dataset into two cohorts; a ‘super healthy’ cohort for fine-tuning and healthy holdout controls, and an ‘at-risk’ cohort for analysis purposes. For the ‘super healthy’ cohort, we identified individuals who were cognitively unimpaired using the Clinical Dementia Rating (CDR) and the Mini-Mental State Examination (MMSE). The CDR score is based on a scale of 0–3; no dementia (CDR = 0), questionable dementia (CDR = 0.5), mild cognitive impairment (CDR = 1), moderate cognitive impairment (CDR = 2), and severe cognitive impairment (CDR = 3) [26]. The MMSE score is based on a scale of 30–0; no dementia (MMSE = 30), questionable dementia (MMSE = 26–29), mild cognitive impairment (MMSE = 21–25), moderate cognitive impairment (MMSE = 11–20), and severe cognitive impairment (MMSE = 0–10) [27]. The super healthy cohort comprised individuals with MMSE > 28 and CDR = 0. We further restricted this cohort to individuals who did not carry an Apolipoprotein E (ApoE) ε4 allele, a major common genetic risk factor for AD. We randomly split the super healthy cohort into two sets: data from 169 individuals was used for fine-tuning and the remaining 168 individuals constituted the healthy holdout dataset. Where individuals had data from more than one visit, we used the data from the baseline visit. The ‘at-risk’ cohort consisted of the remaining 592 individuals. Demographic and AD biomarker information for the super healthy cohort and ‘at-risk’ cohort, for the latest visit, is given in Table 1. The same T1 and dMRI features as for the UK Biobank were extracted for the EPAD dataset, processed using the pipelines described by Lorenzini et al. [28]. There are several differences in image acquisition and processing between the EPAD and UK Biobank datasets, including the scanners used, the choice of processing procedures, the software employed, and the FreeSurfer versions used to generate brain ROIs. As such, we fine-tune our normative model on a cohort of healthy controls from the EPAD dataset. AD CSF biomarkers used for statistical analysis included: phosphorated tau (p-tau) level, total tau (t-tau) level, amyloid-beta 42 (abeta42) level, and p-tau/abeta42 ratio [29]. These biomarkers were quantified using a harmonized pre-analytical protocol with analyses being performed with a fully automatized Roche cobas Elecsys System at the Clinical Neurochemistry Laboratory, Mölndal, Sweden [24]. Concentrations of abeta42 were determined using the manufacturer’s guidelines.

**Table 1:** EPAD demographics

	Super healthy (baseline)	'at-risk'(baseline)	'at-risk'(latest visit)
N	337	592	592
Sex (M:F)	199:138	351:241	351:241
Mean ± sd Age (years)	64.0 ± 7.0	65.4 ± 7.6	65.6 ± 7.6
Mean ± sd ICV	1.50 ± 0.15	1.49 ± 0.16	1.49 ± 0.16
Mean ± sd p-tau pg/mL CSF	16.7 ± 7.32	20.3 ± 9.96	20.3 ± 9.34
Mean ± sd t-tau pg/mL CSF	198 ± 74.0	231 ± 94.3	231 ± 92.2
Mean ± sd abeta42 pg/mL CSF	1555 ± 797.1	1310 ± 692.2	1353 ± 717.1
Mean ± sd CDR global score	0	0.19 ± 0.25	0.19 ± 0.25
Mean ± sd MMSE total score	29.6 ± 0.48	28.1 ± 1.80	28.2 ± 1.77
ApoE (0 variants:1 variant:2 variants)	325:0:0	259:252:32	272:252:32

To adjust for confounding effects, we removed non-linear age (using thin plate regression splines [30]) and linear intracranial volume (ICV) and sex effects from the dMRI and T1 MRI features of both datasets separately. For the EPAD dataset, we used the fine-tuning set to train the regression model and applied the trained model to the holdout and test cohorts. Each brain ROI was normalised by removing the mean and dividing by the standard deviation of the healthy control cohort brain regions effectively resulting in Z-scores.

### Normative modelling framework

**Fig. 1:**
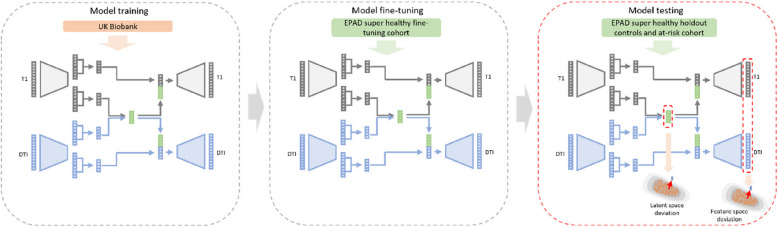
Schematic overview highlighting which dataset cohorts are used in each stage of the modelling process. The left panel shows the training of the entire network using the UK Biobank healthy control cohort. The middle panel shows fine-tuning of the model weights using the EPAD super healthy fine-tuning control cohort in a transfer learning approach. The right panel shows the EPAD super healthy holdout and at-risk cohorts being passed through the network and the resultant latent vectors and data reconstructions being used to calculate individual-level deviations

In this work, we use the disentangled multimodal VAE (DMVAE) autoencoder proposed by Lee and Pavlovic [23] implemented as part of the multi-view-AE Python package [31]. The DMVAE model consists of separate encoder and decoder networks per modality with a shared latent space as well as private latent variables. A schematic of the DMVAE model is shown in Supplementary Figure S1. To use DMVAE as a normative model, the encoder and decoder parameters are trained to characterise a healthy population cohort (i.e., UKB). Autoencoder-based normative models assume abnormality due to disease effects can be quantified by measuring deviations in the latent space [21] or in the feature space [32]. At test time, the clinical cohort is passed through the encoder and decoder networks. Deviations of test subjects from the multi-modal latent space of the healthy controls and data reconstruction errors are measured providing an aggregate measure of abnormity across brain regions and imaging modalities and per region abnormality measures, respectively. The model parameters are given in the Supplementary Material. A schematic overview of the modelling process is given in Fig. 1.

In this work, we used two different z-score measures of deviation: a single aggregate metric and regional deviation metrics. Aggregate deviation metrics are useful for deriving a sensitive, single generic measure of abnormality which could be used to flag individuals which require closer inspection. In contrast, regional metrics can prove useful for a more fine-grained or disease-specific exploration of deviations, for example to create maps which provide an interpretable image of which brain regions show abnormalities. In this work, the aggregate deviation metric, which we term D_ml_, measures the deviation from the multi-modal latent space of the autoencoder-based deep normative model. Here, “ml” refers to a *multivariate latent* space metric. Regional metrics, D_uf_, which measure deviation in each brain imaging ROI, are derived in the feature space by comparing the autoencoder reconstruction to the original input. Here, “uf” refers to a *univariate feature* space metric. The formulation of both metrics, previously introduced by Lawry Aguila, Chapman, and Altmann [19], is provided in the Supplementary Material.

### Statistical analysis

To assess the extent to which general deviation metrics correlated with AD-related biomarkers and cognitive performance, we calculated D_ml_ for the super healthy holdout cohort and the ‘at risk’ cohort. Where data from multiple visits was available for the ‘at risk’ set, we used the data from the most recent visit. To explore AD-specific deviations we extracted the D_uf_ values for the left and right Hippocampus, two AD related brain regions previously identified in the literature [33]. For both D_ml_ and D_uf_ metrics, deviation values are calculated relative to the holdout control set to account for any deviation resulting from suboptimal model fit. Both types of metrics were correlated with the age-adjusted AD biomarkers and cognitive scores. The imaging and biomarker data were taken from the same visit. To adjust for age-related effects, we regressed out the effects of age, using regression coefficients learnt using the super healthy cohort, from each of the biomarkers.

To explore heterogeneity in cohorts stratified by cognitive ability, we calculated, for each brain region, the proportion of outliers in the ‘at-risk’ test cohort using D_uf_. For T1 and FA features we used an outlier cut-off of D_uf_ < −1.96 (i.e., a 2.5% threshold) and for MD features we used a cut-off of D_uf_ > 1.96 to reflect the expected direction of change in these features associated with AD. D_uf_ values below, or above, these thresholds were considered outliers. We stratified the ‘at-risk’ test cohort by CDR and MMSE. For CDR stratified groups, we created outlier maps for CDR = 0 and CDR = 0.5. We also created outlier maps for the following MMSE subgroups; MMSE = 30 (no dementia), MMSE = 28–29 (questionable dementia), MMSE = 26–27 (questionable dementia), MMSE = 21–25 (MCI).

We then explored grey matter heterogeneity in MMSE subgroups at a network level. Following a similar approach to Segal et al. [34], we assigned each cortical region to one of seven functional cortical networks using the Yeo network parcellation [35]. The subcortical regions were assigned to the following groups; medial temporal lobe (Amygdala and Hippocampus), Thalamus or Basal Ganglia (Nucleus Accumbens, Pallidum, Putamen and Caudate nucleus). For each network, we calculated the proportion of individuals within each subgroup where D_uf_ surpassed the specified threshold in at least one region assigned to that network. To quantify the significance of these outlier proportions, we conducted group-wise permutation tests to obtain p-values for each network. The purpose of the permutation tests is to quantify the significance of the proportion of outliers relative to the healthy holdout data. For each subgroup, we permuted the group labels (cases and controls) and repeated the process 10,000 times to derive an empirical distribution of outlier maps under the null hypothesis of random group assignment. Using the D_uf_ calculated outlier proportions, we can obtain p-values from the proportion of null values that exceeded the observed outlier proportion for each brain region. Statistically significant effects were identified using an FDR-corrected [36] threshold of *p* < 0.05.

To explore differences in subgroups stratified by genetic risk, we computed D_ml_ and D_uf_ using imaging data from the baseline visit for the ‘at-risk’ test cohort, where all participants were deemed cognitively normal. We stratified D_uf_ by these cohorts of genetic risk and calculated Cohen's d effect sizes for each region between the healthy holdout cohort and each subgroup. Positive effect size values correspond to subgroups having a higher D_uf_ compared to controls, while negative effect size correspond to subgroups having lower values relative to controls. The genetic subgroups consisted of individuals with; wild-type ApoE (ε3/ε3), one ε4 allele (ε3/ε4) and ε4 homozygous (ε4/ε4).

To explore how general measures of deviation changed over time, for a sub-group of the ‘super healthy’ and ‘at-risk’ cohorts for which we have the required data, we calculated D_ml_ from the baseline and 12-month imaging data. To assess the separation between groups for each cohort, we conducted paired t-tests.

## Results

### Adjusting for confounding effects

Example UMAP plots of the imaging features before and after adjusting for confound effects are given in Supplementary Figures S2 and S3. The association between the UMAP vectors and confound variable was tested using linear regression. We see some residual age effect for the holdout and test cohort, likely due to a combination of disease effect and scans from multiple visits being present in the data. The residual age effects for the holdout and test cohort are further decreased for the D_uf_ values (see Supplementary Figure S4).

### Relationship between deviation metrics and AD biomarkers and cognitive measures

Table 2 shows the Pearson correlation coefficients, and corresponding *p*-values, between D_ml_ for the at-risk cohort and age-adjusted AD biomarkers and cognitive tests results. We see statistically significant associations (Bonferroni adjusted *p*-value threshold = 0.0083) for CDR global score, MMSE total score, abeta42, and p-tau/abeta42 ratio. Next, we looked at the correlations between D_uf_ and age-adjusted AD biomarkers and cognitive scores for the left and right Hippocampus (Table 3). In these regions, we generally see higher correlation between deviation metrics and AD metrics than observed for the general deviation metric, D_ml_. We additionally provide correlations between hippocampal volume features and AD biomarkers in the Supplementary Table S5. Compared to age, sex and ICV adjusted hippocampal volume, D_uf_ of the hippocampus shows stronger correlations with CSF tau biomarkers as well as cognition (CDR, MMSE).

**Table 2:** Correlation results for D_ml_ for the EPAD test cohort

D_ml_	N	Correlation	*P*-value
p-tau	378	0.099	0.054
t-tau	378	0.074	0.150
abeta42	378	−0.145	**4.72e-03**
p-tau/abeta42	378	0.203	**6.93e-05**
CDR global score	587	0.177	**1.58e-05**
MMSE total score	445	−0.128	**6.71e-03**

Pearson correlation coefficient and *p*-value between AD biomarkers or cognitive scores and D_ml_ for the EPAD test cohort. Bold font indicates statistically significant results

**Table 3:** Correlation results for D_uf_ for the left Hippocampus and right Hippocampus

	D_uf_ Hippocampus	Correlation	*P*-value
Left	p-tau	−0.192	1.73e-04
	t-tau	−0.187	2.57e-04
	abeta42	0.170	8.80e-04
	p-tau/abeta42	−0.260	3.02e-07
	CDR global score	−0.216	1.31e-07
	MMSE total score	0.197	2.83e-05
Right	p-tau	−0.202	7.59e-05
	t-tau	−0.193	1.55e-04
	abeta42	0.104	0.043
	p-tau/abeta42	−0.235	3.70e-06
	CDR global score	−0.203	6.96e-07
	MMSE total score	0.189	6.04e-05

Pearson correlation coefficient and *p*-value between AD biomarkers or cognitive scores and D_uf_ for the left Hippocampus and right Hippocampus. Note that the direction of correlation is reversed compared to D_ml_ as here a more negative D_uf_ indicates a greater deviation. Sample sizes are listed in Table 2

### Stratifying deviations by cognitive ability

**Fig. 2:**
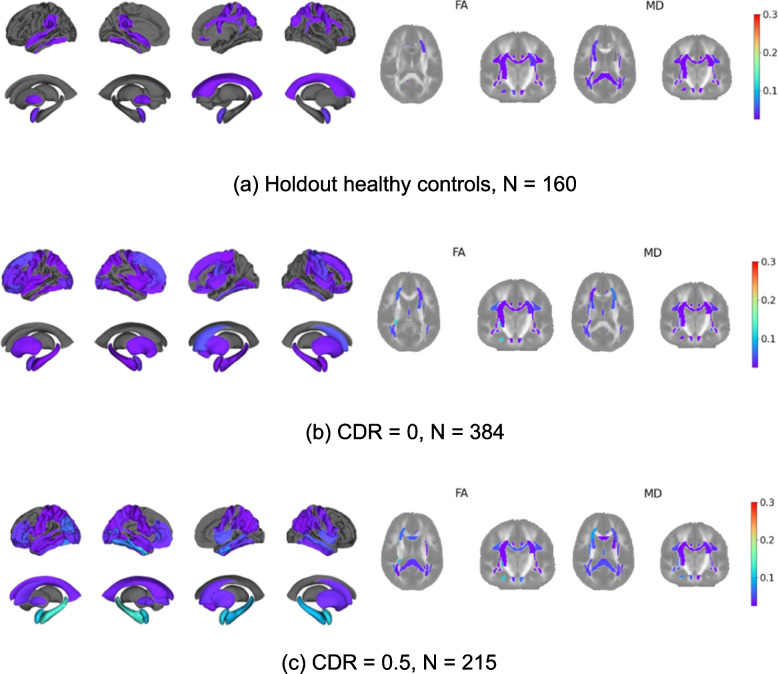
Proportion of regional outliers for the holdout cohort and CDR score subgroups. The colour bar reflects the proportion of outliers from 0.025 to 0.3. Regions in grey indicate that the proportion of outliers for that region was less than 0.025

**Fig. 3:**
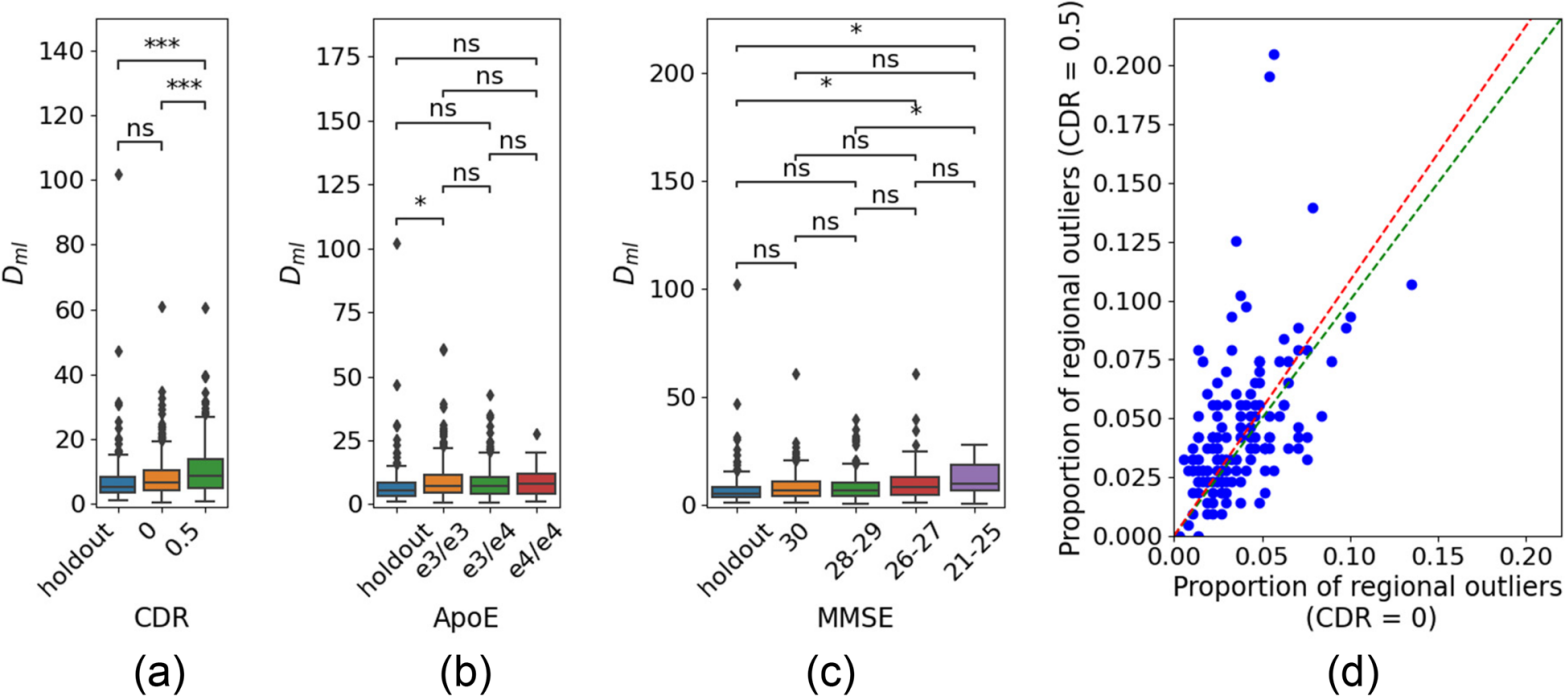
D_ml_ for the holdout cohort and test cohort stratified by (**a**) CDR score (holdout; *N* = 160, CDR = 0; *N* = 384, CDR = 0.5; *N* = 215), (**b**) number of ApoE variants and (holdout; *N* = 160, ε3/ε3; *N* = 224, ε3/ε4; *N* = 252, ε4/ε4; *N* = 32), (**c**) MMSE score (holdout; *N* = 160, MMSE = 30; *N* = 119, MMSE = 28–29; *N* = 208, MMSE = 26–27; *N* = 88, MMSE = 21–25; *N *= 29). The statistical annotations were generated using Welch's t-tests between groups; ns: 0.05 < *p* < = 1, *: 0.01 < *p* < = 0.05, **: 0.001 < *p* < = 0.01, ***: 0.0001 < *p* < = 0.001, ****:*p* < = 0.0001. (d) Each dot represents one imaging derived brain phenotype and its proportion of outliers in the CDR = 0 subset (x-axis) and CDR = 0.5 subset (y-axis). The red-dotted line marks a linear regression between the two cohorts, where a gradient greater than 1 (dotted green line) indicates that the proportion of outliers for CDR = 0.5 is generally greater than the proportion of outliers for CDR = 0

We created proportion of outlier maps, using the D_ul_ deviation thresholds discussed in the statistical analysis section, stratified by CDR and MMSE. Figure 2 shows the proportion of outliers for the healthy holdout cohort, and individuals with a CDR global score of 0 or 0.5. We see little difference between the holdout and CDR = 0 outlier maps. However, we see a higher proportion of outliers for the CDR = 0.5 cohort, in line with the observation of higher D_ml_ with increasing CDR score (refer to Fig. 3a). Figure 3d further illustrates the difference in the proportion of outliers between the CDR = 0 and CDR = 0.5 cohorts, with the red dotted line indicating an overall higher proportion of outliers for CDR = 0.5.

**Fig. 4:**
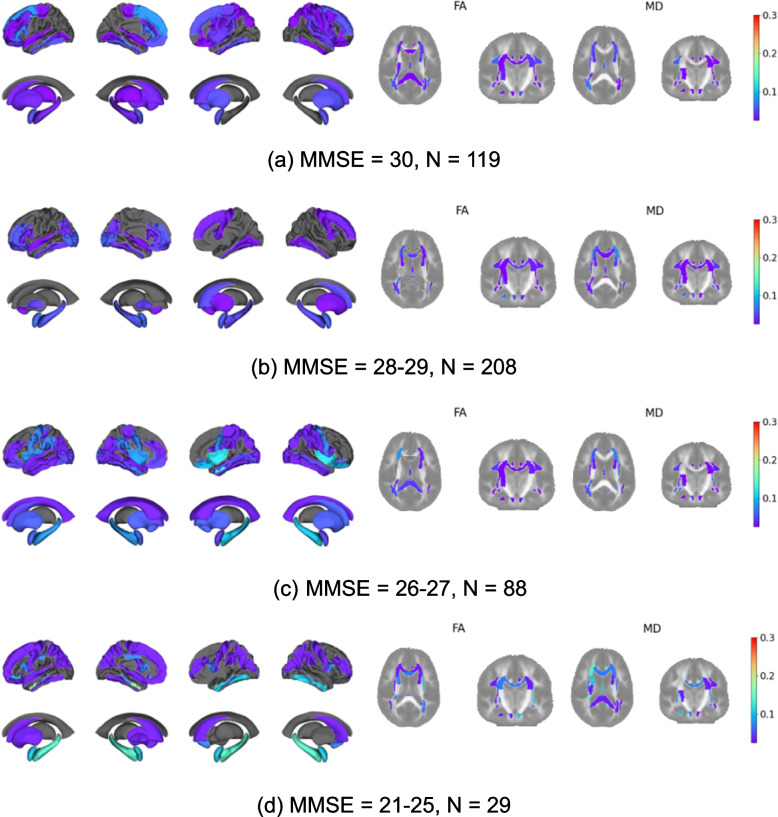
Proportion of regional outliers for the MMSE score subgroups. The questionable dementia subgroup was split into a further two subgroups of MMSE = 26–27 and MMSE = 28–29. The cognitively impaired subgroup ranged from MMSE = 21–25. The colour bar reflects the proportion of outliers from 0.025 to 0.3. Regions in grey indicate that the proportion of outliers for that region was less than 0.025

Figure 4 shows the proportion of outliers for the MMSE subgroups. We see increasing proportion of outliers with decreasing MMSE score, again in the T1 temporal regions and several MD features. Conversely, fewer FA regions show much difference in the proportions of outliers across MMSE subgroups.

### Mapping neural heterogeneity at a network level

**Fig. 5:**
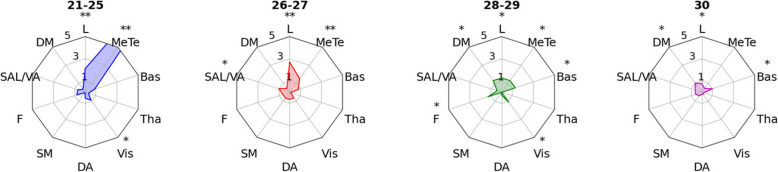
The network level -log_10_ *p*-values associated with the proportion of outliers for each MMSE subgroup under group-based permutation testing. MMSE = 30: *N* = 119; MMSE = 28–29: *N* = 208; MMSE = 26–27: *N* = 88; MMSE = 21–25: *N* = 29. ** corresponds to p_FDR_ < 0.05 and * corresponds to p_uncorrected_ < 0.05. L = limbic, DM = default mode, SAL/VA = salience–ventral attention, F = frontoparietal, SM = somatomotor, DA = dorsal attention, Vis = visual, Tha = thalamus, Bas = basal ganglia, MeTe = medial temporal lobe

The network level -log_10_ *p*-values are given in Fig. 5. The participant groups with the strongest cognitive impairment also exhibited the strongest associations on network level. With decreasing cognitive performance, we see increasingly significant *p*-values obtained from the permutation tests. Moreover, the medial temporal lobe is implicated for the MMSE = 21–25 and MMSE = 26–27 subgroups. In addition, for these two subgroups the limbic network is also implicated. Overall, over half the network regions are implicated (p_uncorrected_ < 0.05) in the MMSE = 28–29 subgroup, however, the *p*-values fail to withstand FDR correction.

###  Deep Normative model identifies genetic risk

We investigated whether the deep normative model could discern differences among cohorts with varying genetic risks for AD. Figure 3b shows D_ml_ for the EPAD cohorts stratified by genetic risk. We found no statistically significance differences in D_ml_ between the holdout cohort (individuals with ε3/ε3 among other restrictions), individuals with ε3/ε3, ε3/ε4 and ε4/ε4.

**Fig. 6:**
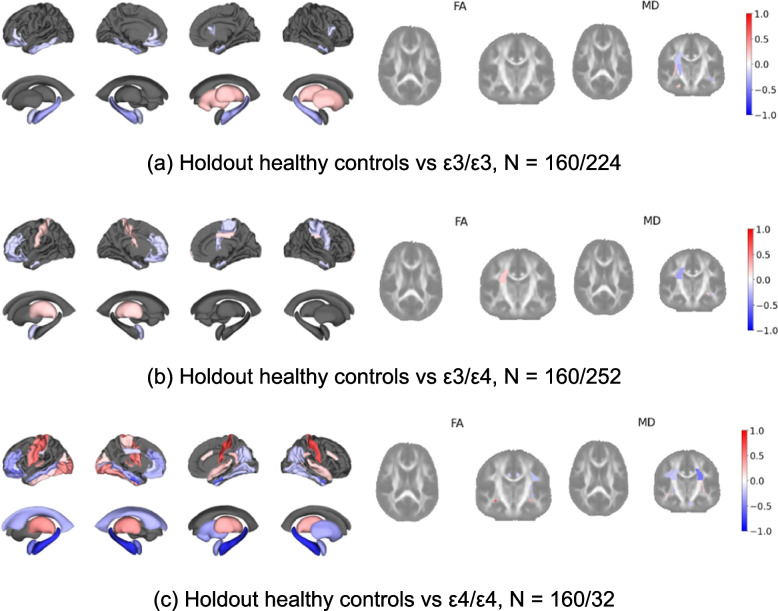
Cohen's d effect sizes between healthy holdout and subgroups of genetic risk using D_uf_. Regions in grey indicate an effect size of −0.2 < d < 0.2

Figure 6 shows the Cohen's d effect sizes for each region between the healthy holdout cohort and each subgroup. Notably, the ε4/ε4 subgroup displays the most substantial negative effect sizes, particularly in the right Hippocampus (d = −0.897), left Hippocampus (d = −0.860), left (d = −0.709), and right Entorhinal cortex (d = −0.690). The direction of effect corresponds to more negative D_uf_ relative to healthy controls. There are large effect sizes for a number of other grey and white matter regions, namely; right Postcentral (d = 0.735), right FX/ST FA value (d = 0.717), and left Postcentral (d = 0.608).

###  Latent deviation reflects longitudinal change

**Fig. 7:**
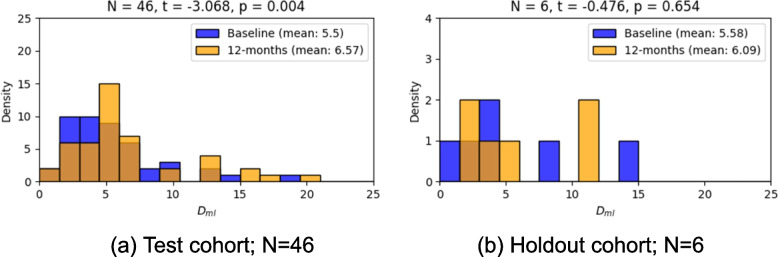
Histogram of D_ml_ for visits at baseline and 12-months for the (**a**) test cohort and (**b**) holdout cohort. We conducted pairwise t-tests between baseline and 12-month visits for each cohort with the t-statistic and *p*-value provided in the title of each subplot

For both the test cohort and the healthy holdout cohort, we identified individuals for which we had imaging data for a baseline and follow up visit after 12 months. Paired t-tests of D_ml_ for baseline and 12-month visits showed statistically significantly higher deviations at the follow up visit for the test cohort but not the healthy holdout cohort (Fig. 7).

## Discussion

In this work we applied a deep normative model, trained on the UK Biobank dataset, to the external EPAD dataset with the aim of detecting subtle differences in brain morphology among ‘at-risk’ individuals. We found that single aggregate deviations (D_ml_), which summarize the deviation in whole brain pattern from a healthy normative population, correlated significantly with AD biomarkers and cognitive scores. It is promising that the general metric D_ml_ can already detect subtle differences in AD-specific biomarkers in non-demented individuals. This implies that, without incorporating additional prior knowledge of AD, such as concentrating solely on brain regions associated with AD, the deviation metric effectively identifies dementia-related differences among individuals. This suggests that generic, disease-agnostic normative models together with such deviation metrics can detect subtle morphological changes already occurring in ‘at-risk’ individuals, which could be informative for early diagnosis of AD and other neurodegenerative disorders. Next, we explored the correlations for AD-specific deviation metrics by focusing on the bilateral hippocampi and generally saw much stronger correlations between the deviation metrics and AD biomarkers than observed for general deviation metrics. This highlights the ability of the deep normative model, which had been trained on healthy subjects, to exhibit increased sensitivity when focusing on brain regions that are susceptible to disease effects. These results allude to the potential for first using aggregate deviation metrics to flag outlying individuals before exploring disease specific deviations.

To provide insights into the normative model, we explored feature space deviations and derived outlier maps stratified by cognitive scores. We sought to identify whether the brain regions highlighted by the normative model as abnormal were specific to AD. We found that generally the proportion of outliers per brain region increased with decreased cognitive performance as we would expect for people at risk of developing AD. Moreover, once severity of the cognitive decline increased, the brain regions typically associated with AD pathology featured the most outliers. For instance, when stratifying the test cohort by CDR score, we saw little difference between the holdout and CDR = 0 outlier maps, aligning with expectations since a CDR score of 0 is considered cognitively normal, and thus any abnormality potentially present is minimal at this point and challenging to detect using T1w MRI or DTI (Fig. 2b). However, we see a higher proportion of outliers for the CDR = 0.5 cohort, particularly in the temporal region, consistent with findings from previous AD studies (Fig. 2c) [37]. This suggests that the deep normative model effectively detects subtle differences between these cohorts. For the MMSE stratified groups, we saw an increasing proportion of outliers with decreasing MMSE score particularly in the T1 temporal regions and several MD features. Fewer FA features showed pronounced difference in the proportion of outliers across the MMSE subgroups, a finding consistent with previous studies suggesting that FA is less sensitive to group differences in AD [14]. In general, regions with the highest outlier counts corresponded to those highlighted in previous AD studies, and results were generally in line with previous AD normative analysis [37].

Exploring the grey matter deviations at a network level, permutation tests found statistically significant *p*-values for the questionable dementia and MCI MMSE subgroups in network regions associated with the early stages of AD, namely the medial temporal lobe and limbic region. The medial temporal lobe is home to the amygdala and hippocampus, previously linked to AD [11]. It is well known that the limbic system is involved with AD and atrophy in these brain regions is one of the earliest hallmarks of the disease [38].

Previous analyses focused on cognitive decline. However, we also investigated deviations stratified by AD genetic risk, particularly the ApoE ε4 allele. For the general measure of deviation (D_ml_) there were no statistically significance differences between the holdout cohort, individuals with ApoE ε3/ε3, one ε4 allele and ε4/ε4. This lack of significance is not entirely unexpected, since our focus was solely on genetic risk which does not have to definitively indicate abnormality. Furthermore, considering we used the baseline measurements, any potential abnormalities in the imaging data may be too subtle and narrowly distributed across regions to be detected by a general measure of abnormality. By contrast, for the regional level deviations, we found that ε4 homozygous individuals exhibited shrinkage relative to healthy controls in some temporal regions. It is particularly interesting that we can observe these differences at baseline, where all individuals are considered cognitively normal, precluding any clinical diagnosis of AD. Interestingly, in this analysis we also observed several grey and white matter regions which appear to show growth.

Lastly, we examined how latent deviations evolved over time for our ‘at-risk’ test cohort and healthy holdout cohort. We found there was a statistically significant increase in latent deviation between the baseline and 12-month visits for the test cohort, a trend not observed in the healthy holdout cohort. This is promising as it suggests that the deviation in our healthy holdout cohort remains stable across visits, with no deterioration in grey or white matter regions. Conversely, the increasing deviation in the test cohort aligns with expectations for a group with either genetic risk or borderline cognitive scores. This suggests an increase in abnormality in the ‘at-risk’ cohort, possibly associated with early-stage AD. It is promising that we observe differences in D_ml_ in such a short timeframe.

This study has several limitations. Firstly, it should be noted that we did not exclude ε4 carriers from the UK Biobank training cohort. As such, preclinical neurodegeneration from genetic risk may be present as part of the normative model. Secondly, subgroups within the EPAD dataset varied in size and the availability of longitudinal samples with all the required imaging and biomarker data was limited, which may limit the robustness of the results. To address this, future research efforts should prioritise the application of deep normative modelling approaches to large longitudinal cohorts focusing on ageing and the early stages of AD. Furthermore, AD biomarkers were not used to define the super healthy cohort and so some subjects may already have amyloid deposition. Another limitation is that the longitudinal analysis was limited to just two visits. Further work could involve comparing the longitudinal trajectories observed here to other AD datasets with a larger number of timepoints, such as the ADNI dataset [39]. This could offer a clearer understanding of how deviation measures can effectively monitor cognitive decline over time. Finally, the EPAD data was collected from multiple centres, which could introduce site-related effects as potential confounders. However, the data acquisition followed a standardized scanning protocol designed to minimize between-site differences while accounting for variations in scanner hardware and software limitations [28]. Additionally, any remaining site effects could be mitigated using harmonization methods such as NeuroComBat [40].

In conclusion, in this work, we have showcased the ability of deep normative modelling approaches to detect subtle differences in brain morphology in individuals at risk of developing AD in the EPAD dataset. The findings presented here, which align with previous AD research, highlight the potential of deep normative modelling for individual-level analysis of the early stages of AD. Furthermore, as the normative model is not specifically trained for AD, there is potential to adapt the model to other neurodegenerative diseases.

## Supplementary Information

Supplementary Material 1

## Data Availability

No datasets were generated or analysed during the current study.

## Abbreviations

AD: Alzheimer's disease
ADNI: Alzheimer’s Disease Neuroimaging Initiative
CDR: Clinical Dementia Rating
DTI: Diffusion tensor imaging
EPAD: European Prevention of Alzheimer’s Dementia
FA: Fractional Anisotropy
MD: Mean Diffusivity
MMSE: Mini-Mental State Examination
UKB: UK Biobank
