# Supplementary material for: Deep normative modelling reveals insights into early-stage Alzheimer's disease using multi-modal neuroimaging data
Source: Alzheimers Res Ther. 2025 May 15;17:107. doi: 10.1186/s13195-025-01753-3 (PMC12080058; doi:10.1186/s13195-025-01753-3)
Supplement: Supplementary file 1 — Supplementary Material 1 [file 13195_2025_1753_MOESM1_ESM.docx]

**Deep normative modelling reveals insights into early-stage Alzheimer's disease using multi-modal neuroimaging data**

Ana Lawry Aguila^1^, Luigi Lorenzini^2,3^, Mohammed Janahi^1,4^, Frederik Barkhof^1,2,5^, Andre Altmann^1^

Affiliations

1 UCL Hawkes Institute, Department of Medical Physics and Biomedical Engineering, University College London (UCL), London, UK

2 Department of Radiology and Nuclear Medicine, Amsterdam University Medical Center, Amsterdam, 1081 HV, The Netherlands

3 Amsterdam Neuroscience, Brain Imaging, Amsterdam, the Netherlands

4 Medical and Population Genomics Lab, Human Genetics Department, Research Branch, Sidra Medicine, Qatar

5 UCL Queen Square Institute of Neurology, University College London, London, WC1N 3BG, UK

Corresponding Author:

Ana Lawry Aguila, ana.aguila.18@ucl.ac.uk and Andre Altmann, a.altmann@ucl.ac.uk

Current address: Athinoula A. Martinos Center for Biomedical Imaging, Harvard Medical School and Massachusetts General Hospital

**Supplementary Material**

**Deep normative modelling framework**


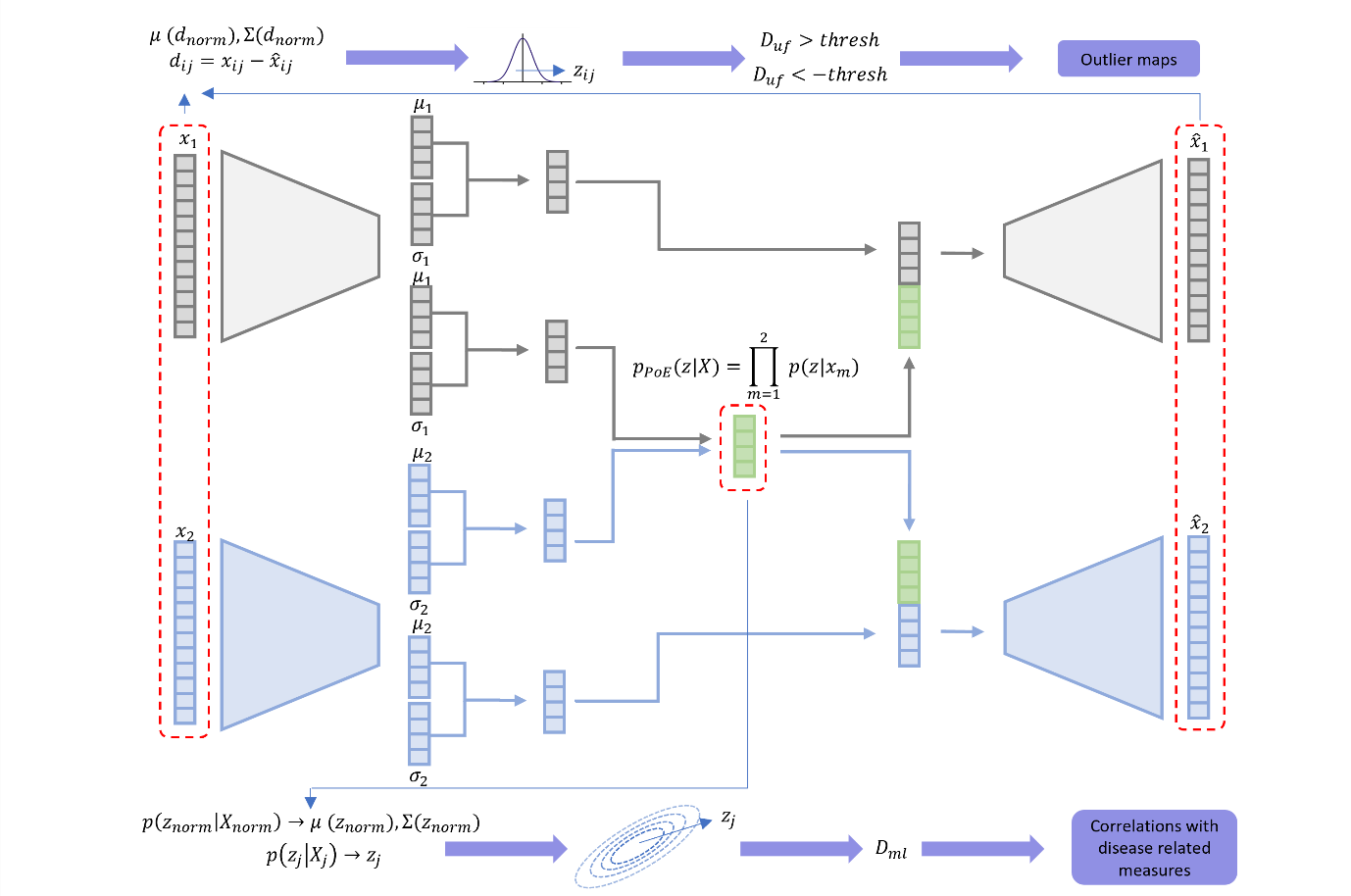
 **Figure S1:** DMVAE model and deep normative modelling framework.

The DMVAE model was implemented with the following parameters optimized through a parameter screening (1); z_shared_=5, z_private_=15, β=1, maximum epochs=2000, batch size=256, learning rate=10^-4^, early stopping=50 epochs, encoder layers=[20, 40], decoder layers=[20, 40]. A ReLU activation function was applied between layers.

***Deviation metrics***

In this section, we provide the formulation for the deviation metrics $D_{ml}$ and $D_{uf}$. Latent and feature space deviation metrics can provide distinct insight into abnormality across disease groups. Previous work [26,10,13] has quantified feature space deviation metrics as the squared-error between the original data and its reconstruction:

$$d_{ij}={(\hat{x}_{ij}-x_{ij})}^{2}$$

where $x_{ij}$ is the input for i-th brain region of subject j and $\hat{x}_{ij}$ the value reconstructed by the autoencoder. This approach works well when we expect all deviations to be in the same direction or if each region only experiences growth or shrinkage. However, where we expect directional deviations, we risk losing information by using a square-error based metric. Here, we instead opt for the following feature-based deviation metric:

$$d_{ij}=(\hat{x}_{ij}-x_{ij})$$

allowing us to explore positive and negative deviations in the feature space. We normalise the data deviation relative to a healthy control cohort to generate the following z-score metric:

$$D_{uf}=\frac{d_{ij}-\mu_{norm}(d_{ij}^{norm})}{\sigma_{norm}(d_{ij}^{norm})}$$

where $\mu_{norm}(d_{ij}^{norm})$ is the mean and $\sigma_{norm}\left( d_{ij}^{norm} \right)$the standard deviation of the deviations $d_{ij}^{norm}$ of the healthy control cohort.

$D_{ml}$, a single multivariate measure of deviation, measures the Mahalanobis distance (2) from the latent encoding distribution of a healthy cohort:

$$D_{ml}=\sqrt{\left( z_{j}-\mu\left( z^{norm} \right) \right)^{T}{\Sigma\left( z^{norm} \right)}^{-1}(z_{j}-\mu\left( z^{norm} \right))}$$

where $z_{j}\sim p(z_{j}|X_{j})$ is a sample from the joint encoding distribution for subject j, $\mu\left( z^{norm} \right)$ is the mean and $\Sigma\left( z^{norm} \right)$ the covariance of the healthy cohort latent position.

**Demographics information**

**Table S1:** UK Biobank demographics.

| N | Sex (M:F) | Mean ± sd Age (years) | Mean ± sd ICV |
| --- | --- | --- | --- |
| 12844 | 6431:6413 | 63.3 ± 7.4 | 1.44 ± 0.14 |

**Table S2:** EPAD demographics for each ApoE subgroup of the test cohort.

| ApoE | N | Sex (M:F) | Mean ± sd Age (years) | Mean ± sd ICV |
| --- | --- | --- | --- | --- |
| 0 variants (ε3/ε3) | 224 | 91:133 | 66.8 ± 7.5 | 1.49 ± 0.16 |
| 1 variants  (ε3/ε4) | 252 | 99:153 | 64.2 ± 7.3 | 1.48 ± 0.16 |
| 2 variants  (ε4/ε4) | 32 | 17:15 | 65.9 ± 6.0 | 1.56 ± 0.16 |

**Table S3:** EPAD demographics for each CDR subgroup of the test cohort.

| CDR | N | Sex (M:F) | Mean ± sd Age (years) | Mean ± sd ICV |
| --- | --- | --- | --- | --- |
| 0 | 384 | 245:137 | 64.1 ± 7.3 | 1.48 ± 0.16 |
| 0.5 | 215 | 108:107 | 68.1 ± 7.2 | 1.51 ± 0.16 |

**Table S4:** EPAD demographics for each MMSE subgroup of the test cohort.

| MMSE | N | Sex (M:F) | Mean ± sd Age (years) | Mean ± sd ICV |
| --- | --- | --- | --- | --- |
| 30 | 119 | 67:52 | 63.2 ± 7.4 | 1.52 ± 0.17 |
| 28-29 | 208 | 126:82 | 64.6 ± 7.2 | 1.49 ± 0.16 |
| 26-27 | 88 | 46:42 | 67.3 ± 8.2 | 1.48 ± 0.17 |
| 21-25 | 29 | 17:12 | 70.8 ± 7.0 | 1.43 ± 0.19 |

**UMAP plots**


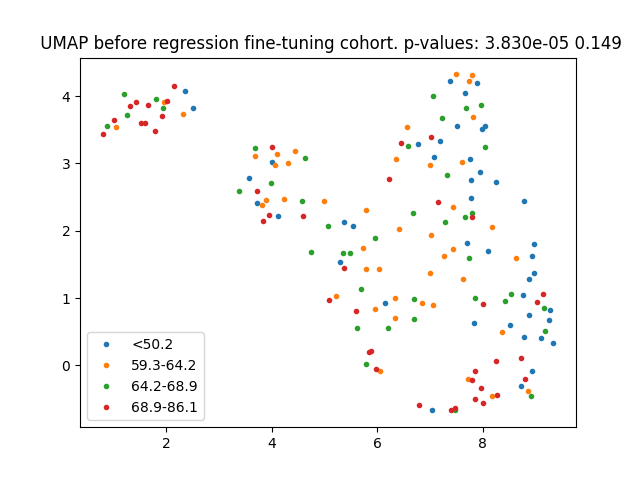

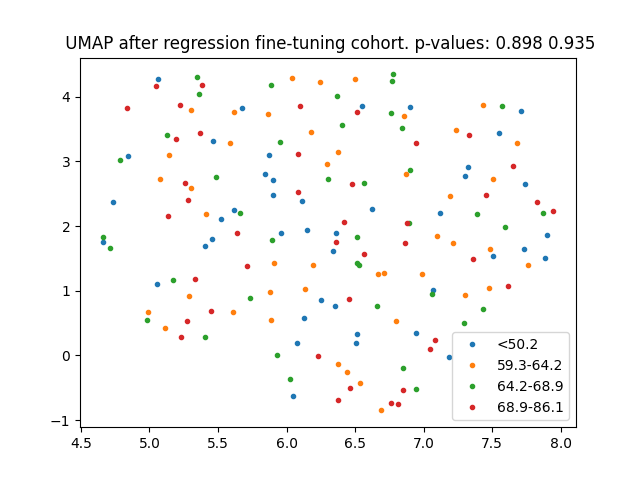


1. (b)


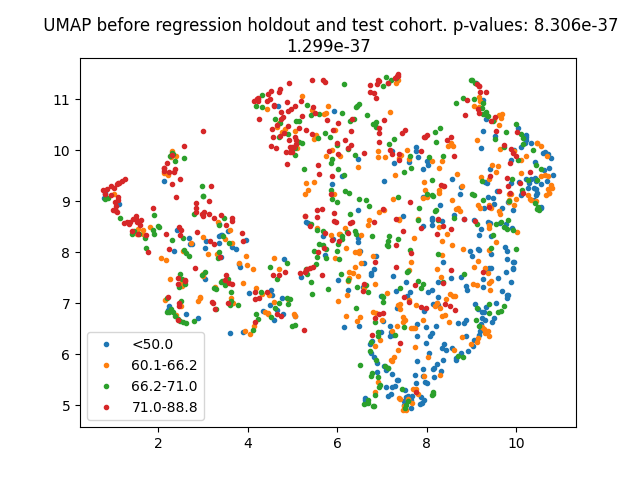

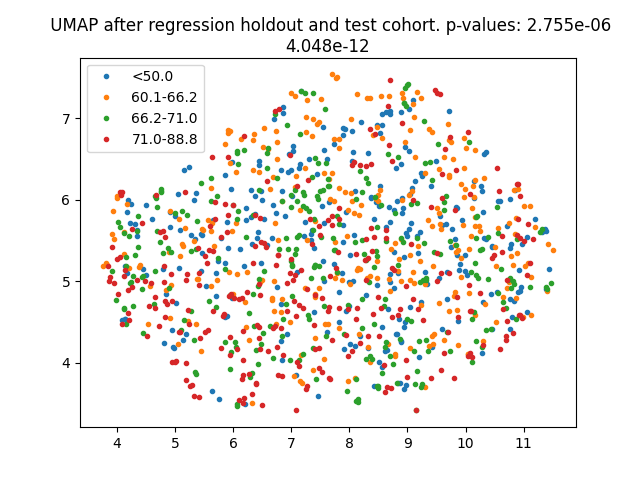


(c) (d)

**Figure S2:** EPAD UMAP plots for T1 features coloured by age for the healthy fine-tuning cohort (a) before and (b) after confound regression, and for the holdout and test cohort (c) before and (d) after confound regression. P-values, calculated using linear regression, are greatly reduced after confound regression.


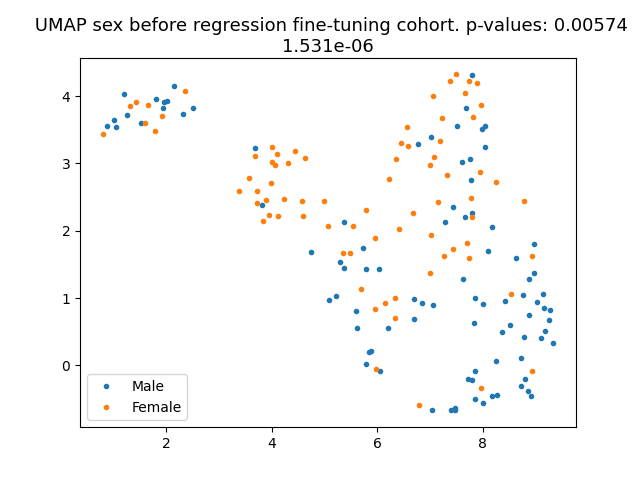

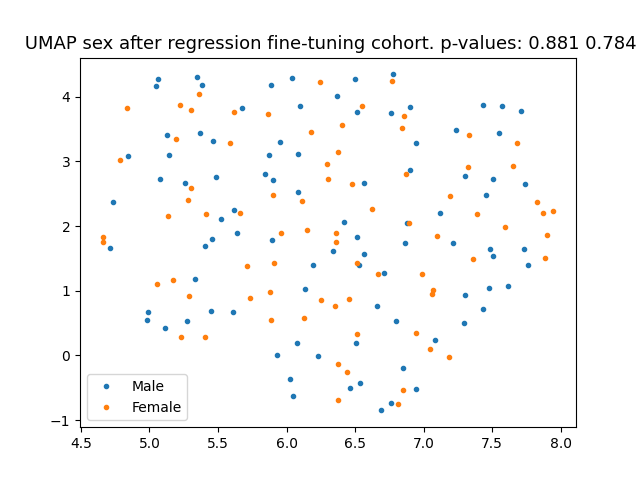


1. (b)


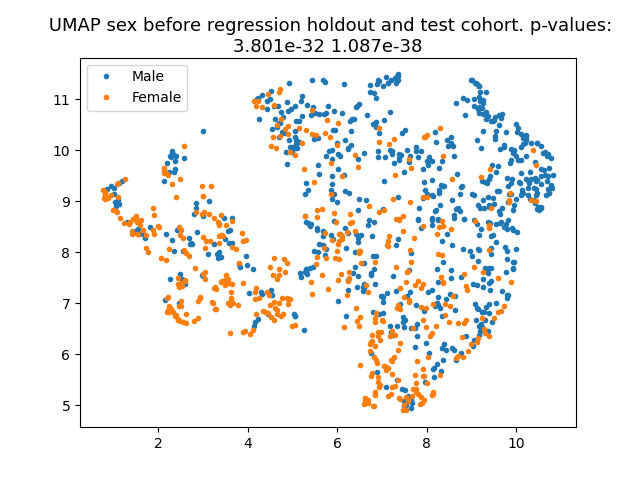

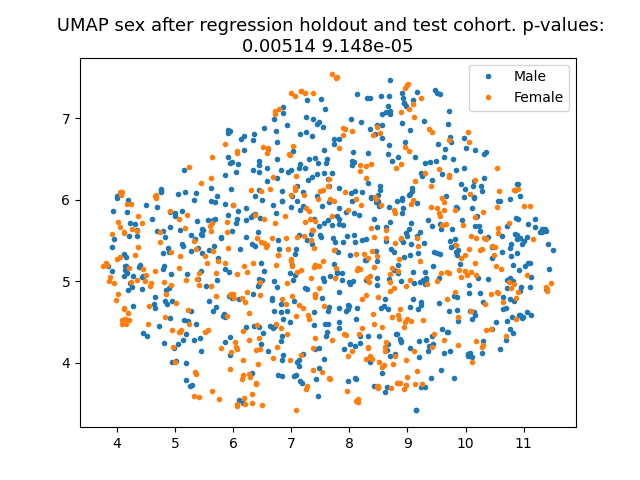


(c) (d)

**Figure S3:** EPAD UMAP plots for T1 features coloured by sex for the healthy fine-tuning cohort (a) before and (b) after confound regression, and for the holdout and test cohort (c) before and (d) after confound regression. P-values, calculated using linear regression, are greatly reduced after confound regression.


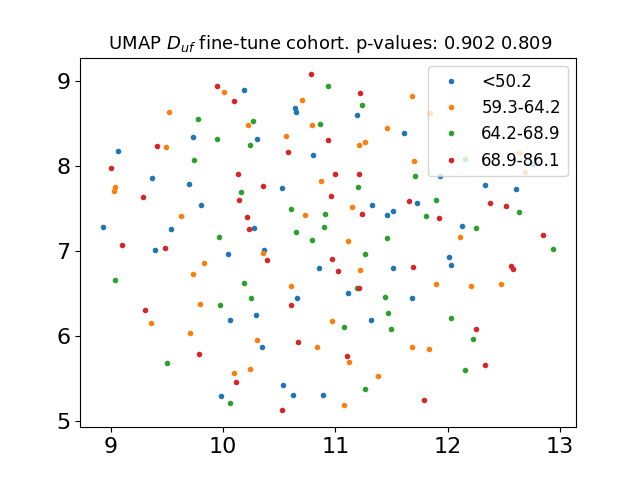

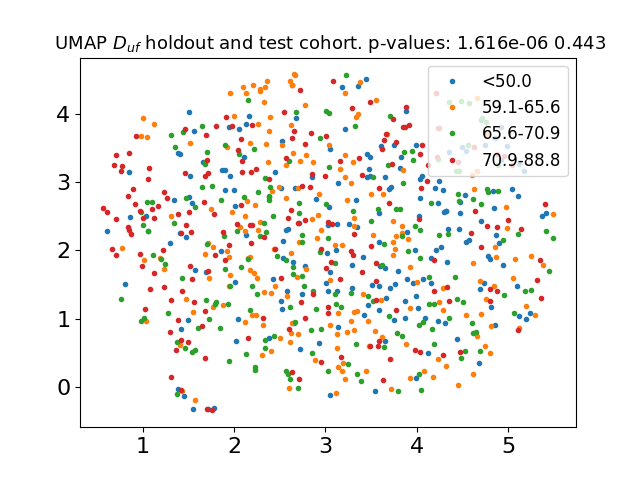


1. (b)


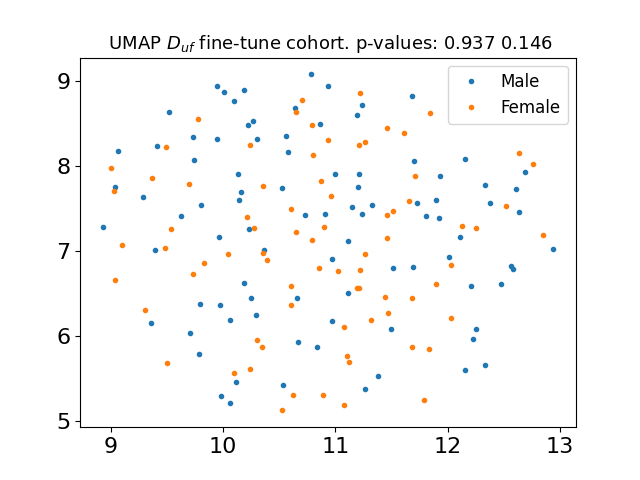

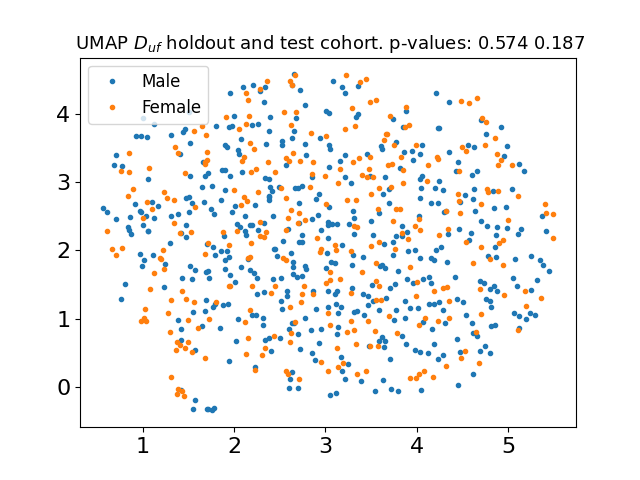


(c) (d)

**Figure S4:** EPAD UMAP plots for D_uf_ T1 features coloured by age for (a) the healthy fine-tuning cohort and (b) for the holdout and test cohort and coloured by sex for (c) the healthy fine-tuning cohort and (d) for the holdout and test cohort. P-values, calculated using linear regression for age and logistic regression for sex, are greatly reduced after confound regression.

**Correlation between raw features and AD biomarkers and cognitive measures**

We followed the same approach as in Table 3 of the main text for the hippocampal volume features (linearly regressing out age, sex and ICV) from the EPAD dataset. Table S5 shows the correlations between age, sex and ICV-adjusted hippocampal volume from the EPAD dataset and age-adjusted AD biomarkers. We see lower correlations and weaker p-values across almost all (10 out of 12) AD biomarkers compared to Table 3. The stronger results for the abeta42 biomarker observed in Table S5 compared to Table 3 could be due to residual confounding effects present in the raw features which are greatly reduced for D_uf_ (see Figure S4b).

**Table S5:** Pearson correlation coefficient and p-value between AD biomarkers or cognitive scores and features for the left Hippocampus and right Hippocampus.

|  | Hippocampal Volume | Correlation | P-value |
| --- | --- | --- | --- |
| Left | p-tau | -0.103 | 0.044 |
|  | t-tau | -0.096 | 0.062 |
|  | abeta42 | 0.201 | 8.31E-05 |
|  | p-tau/abeta42 | -0.244 | 1.59E-06 |
|  | CDR global score | -0.141 | 3.40E-04 |
|  | MMSE total score | 0.149 | 1.54E-03 |
| Right | p-tau | -0.100 | 0.052 |
|  | t-tau | -0.092 | 0.073 |
|  | abeta42 | 0.150 | 3.49E-03 |
|  | p-tau/abeta42 | -0.214 | 2.57E-05 |
|  | CDR global score | -0.135 | 6.42E-04 |
|  | MMSE total score | 0.146 | 1.91E-03 |
